# Arsenic Sold for Antimony

**Published:** 1841-11

**Authors:** 


### ARSENIC SOLD FOR ANTIMONY.—EMPOISONING.

Our friend Prof. J. Warder of Cincinnati College has communicated to us the following fact. A livery-stable keeper in Cincinnati applied to an apothecary for a quantity of what the farriers call antimony, (the well known sulphuret of that metal) and as he supposed obtained it. Administration was made in the usual way by mixing the powder with the provender of his horses. The consequence was that several of them were killed. The symptoms were not noted. A portion of the poison, not exhibited, was brought to the Professor who, on examination, ascertained it to consist of the impure arsenic, commonly called cobalt, or flystone.

Some of the horses were examined after death and exhibited evident traces of gastric inflammation.

We think this a favorable opportunity to put forth a few hints on the state of our establishments for compounding and selling medicines in the West. They are of three classes:—1st. The village and country merchant's dry goods' stores—2nd. The Doctor's offices—3rd. The Druggist's and Apothecaries' shops. The first have grown out of the necessities of a newly settled country ; and are now seldom met with in any of our larger towns. The number of articles kept by merchants is generally few; and they are commonly sold in bulk, except tartar emetic and calomel; and therefore dangerous accidents are not likely to happen. The second class deserve a more extended notice. A student is generally the compounder of prescriptions in a Doctor's office, and no doubt he is *sometimes* qualified for the task, at the time it is confided to him. Such, however, is not always the case. The first study to which he is put, is the bones, when it *should* be the medicines he is practically to work among. Having gone through a tedious and too often unprofitable course of anatomy, he makes a superficial survey of physiology, and then takes up a work on therapeutics, or the practical application of medicines—sometimes even a treatise on the practice of physic! After this, there is a reluctant and unproductive recurrence to chemistry, pharmacy, and the natural history and sensible qualities of medicines—all of which should have been studied before any thing else. On this plan, or rather according to this practice without plan, the student passes through his private pupilage, engaged more or less in compounding the prescriptions of his preceptor, with a most incompetent knowledge of the properties and chemical composition of the articles which he is every day mingling together; and is of course liable to make mistakes. In illustration, we may mention one, which proved fatal. A physician of this state directed a certain quantity of ipecac., or some other medicine, to be mixed with a prescribed quantity of *aqua font.* for a small negro child. Instead of the latter, his student put up *aqua fortis*—the child took it and was killed. Had that young man understood pharmacy and the history and properties of medicinal agents, it is not probable that so serious a mistake could have occurred.

The third class, in most of our cities and larger towns, have, now, in their hands, the preparation and putting up of most of the medicines that are prescribed by physicians; and while the student of medicine generally has the recipe from the mouth of his preceptor and puts it up from memory, the apothecary's clerk has before him the written prescription. On the manner in which this is executed we must say a word. It should be in a legible, open hand, with the name of the patient, the date, and the name or private signature, of the physician, attached to it. How soldom it has these safe and essential requisites, any one may satisfy himself by examining the files of our shops, in Louisville or Cincinnati. If they always had the first, distinct legibility, the matter would not be so bad; but many are so obscurely written, that without a thorough knowledge of medical hieroglyphics, the compounder is liable to fall into egregious blunders. If the physician would arraign the apothecary before the tribunal of public opinion, he should enter it with clean hands. If, as he claims, he is the superior, let him set a good example to his inferior—let him show, on the face of his prescription, that he has thought, at the moment of writing it, of the great necessity of having it correctly understood. He will then have substantial ground to stand upon, in charging the druggist who has fallen into error. Many physicians, moreover, do not read their prescriptions after writing them, by which, mistakes of a formidable kind have passed uncorrected, till their effects were felt by the patient, unless the apothecary rectified them, which has not often happened. While on this prolific subject, we must add a paragraph relative to the language in which prescriptions are written. In the United States where the Latin language is so little cultivated, it is affectation and folly, to write, we should rather say, attempt to write, prescriptions in that language.

Not one in a hundred (any more than ourselves) are capable of writing Latin prescriptions; and if they were thus composed, a still smaller proportion of our apothecarie's clerks would understand them. Moreover, we ask for the advantage of such a practice? We can see none, and wish it were universally abandoned. But we must return to the duties and qualifications of the compounding druggist.

It is the custom of our city apothecaries to take small boys as apprentices and clerks, who are often illiterate, and, always, unacquainted with chemistry and botany, while an elementary knowledge of those sciences, together with a tolerable education, and the age of 15 or 16, should be regarded as indispensable prerequisites. To place a little boy behind the counter of a druggist, as merchants are wont to place boys before their shelves of silks and gridirons, is homicidal; and no physician should sanction it. His own inevitable homicides are more than enough. If he cannot avoid being a principal, he may keep himself from being an accessory, by directing his prescriptions to be sent to shops where none but qualified and careful persons are permitted to handle and put up for use, articles which are the instruments of death not less than of life, when taken in excess. In Europe the apothecary is a man of learning and science—here, too often, he is a mere trader. There, he undergoes a long and laborious apprenticeship—here, he substitutes the probation of weeks, for that of years; and, from the beginning, works among poisons, with as much confidence and unconcern, as a confectioner's apprentice beats up eggs, or licks the syrup from the ends of his fingers. D.

